# An Improved HILIC HPLC-MS/MS Method for the Determination of β-ODAP and Its α Isomer in *Lathyrus sativus*

**DOI:** 10.3390/molecules24173043

**Published:** 2019-08-22

**Authors:** Andreia Bento-Silva, Letice Gonçalves, Elsa Mecha, Filipe Pereira, Maria Carlota Vaz Patto, Maria do Rosário Bronze

**Affiliations:** 1 FFULisboa, Faculdade de Farmácia da Universidade de Lisboa, Avenida das Forças Armadas, 1649-019 Lisboa, Portugal; 2 ITQB NOVA, Instituto de Tecnologia Química e Biológica da Universidade Nova de Lisboa, Avenida da República, Estação Agronómica Nacional, 2780-157 Oeiras, Portugal; 3 FCT NOVA, Faculdade de Ciências e Tecnologia da Universidade Nova de Lisboa, 2829-516 Caparica, Portugal; 4 IBET, Instituto de Biologia Experimental e Tecnológica, Avenida da República, Estação Agronómica Nacional, 2780-157 Oeiras, Portugal

**Keywords:** α-ODAP, β-ODAP, *Lathyrus sativus*, grass pea, HPLC-MS/MS, HILIC column

## Abstract

β-*N*-Oxalyl-l-α,β-diaminopropionic acid (β-ODAP) is a non-protein amino acid present in *Lathyrus sativus* (grass pea) and other *Lathyrus* species, in parallel with its nontoxic isomer, α-ODAP. When consuming grass pea for several months as staple food, β-ODAP may cause neurolathyrism, a motor neuron degeneration syndrome. Therefore, the independent quantification of both ODAP isomers instead of only the total amount in grass pea allows the identification of less toxic varieties and the development of tools to support breeding for improving grass pea quality. In this work, a simple and fast HPLC-MS/MS method was developed without sample derivatization, using a hydrophilic interaction chromatography (HILIC) column and an isocratic gradient of eluents for 18 min, which allowed the determination of both α- and β-ODAP. The proposed method was fully validated and applied to the determination of α- and β-ODAP contents in a diverse collection of 107 grass pea accessions representative of the main grass pea-growing geographical regions in the world, with the prompt identification of contrasting accessions. β-ODAP content in the analyzed grass pea samples ranged from 0.45 ± 0.02 to 6.04 ± 0.45 mg g^−1^. The moderate correlation found between α- and β-ODAP contents (0.65) in this collection reinforces the importance of the independent quantification of both ODAP isomers.

## 1. Introduction

Grass pea (*Lathyrus sativus*) is the most cultivated *Lathyrus* species [1,2], particularly in South Asia and Sub-Saharan Africa [1], and has been used as human food and animal feed since ancient times as an important source of carbohydrates, proteins, and also antioxidant compounds [3]. It is resistant to both flood and drought, and thrives on poor soils that do not support many other crop species growth [2]. However, *Lathyrus* species have been implicated as the cause of a neurological disorder, called neurolathyrism, in both men and animals [2]. Neurolathyrism is characterized by an irreversible neurodegeneration of motor neurons, resulting in a spastic paraparesis of the legs that can vary from mild walking difficulties to bedridden state [4,5,6]. Neurolathyrism is caused by an excessive consumption of grass pea seeds as staple food for several months [5,6] and it is intricately linked to poverty, malnutrition, and periods of famine [2,4,5,7]. Importantly, grass pea is harmless to humans and animals when consumed as part of a balanced diet [1].

The discovery of the neuroexcitatory potential of β-*N*-Oxalyl-l-α,β-diaminopropionic acid (β-ODAP), a non-protein amino acid present in grass pea [7], led to proposing a role as the neurotoxin responsible for the disease [5,6]. The mechanism of its action has not been conclusively elucidated [6]. Nevertheless, potential pharmacological benefits of β-ODAP should not be neglected [1]. The activation of protein kinase C (PKC) by β-ODAP adds a new dimension to explore its possible therapeutic potentials in areas such as Alzheimer’s disease, hypoxia, and long-term potentiation [7]. Interestingly, β-ODAP was also found to be present in the non-leguminous plants *Panax* species, and it is considered a bioactive therapeutic amino acid component in *Panax notoginseng*, commonly referred as dencichine. Studies have reported β-ODAP as the component responsible for the medicinal herb’s main haemostatic and platelet-increasing properties in vivo [8,9]. It has also been described that the haemostatic effect is present at a low dose of β-ODAP, while neurotoxicity occurs at higher doses [8,9].

In *Lathyrus* seeds, β-ODAP is accompanied by lower concentrations of its isomer α-ODAP [2,5]. Unlike β-ODAP, α-ODAP is considered non-toxic, and technological processing of *Lathyrus* seeds or flour, such as cooking, fermentation, or pre-soaking in alkaline solutions, can decrease β-ODAP concentration and increase the concentration of its α isomer, reducing *Lathyrus* toxicity [1,2,10]. Diaminopropionic acid (DAP) can also be formed by steaming, presumably resulting from hydrolysis of the oxalyl compound, decreasing β-ODAP content [8,11].

Given the increasing need for resilient food crops, grass pea breeding has gained importance, and major efforts have been aimed at reducing β-ODAP content, with the development of low β-ODAP varieties through selective breeding [1,2]. High-throughput accurate quantification methods for this specific compound are extremely important for these breeding programs, where a large number of samples need to be quantified in a simple and fast way. Several analytical methodologies have been suggested for β-ODAP quantification, but some were not able to distinguish the toxic β-ODAP from its non-toxic α isomer, such as colorimetric methods [12], GC-MS (gas chromatography-mass spectrometry) after sample derivatization [9] and TLC (thin layer chromatography) [13]. Some other methodologies have been successful in analyzing both α- and β-ODAP, namely by NMR (nuclear magnetic resonance) [14] and CZE (capillary zone electrophoresis). However, the high operation pH (9.2) in CZE may result in β-ODAP hydrolysis to diaminopropionic acid (DAP) [13,15,16].

Although these methods have been employed, HPLC (high performance liquid chromatography) has been the most used for β-ODAP quantification [8,9,12,13,15,17,18,19,20,21]. However, the analysis of ODAP by HPLC is a challenge due to its high polarity and weak UV absorption [8]. The majority of the currently available HPLC based methods for β-ODAP quantification involve the use of a reversed phase C18 column and UV detection, after a multi-step derivatization of the sample [17,18,19,22]. However, poor derivative stability, side reactions, and reagent interferences may occur, which may lead to interference signals [8,13,20]. Furthermore, these methods are usually laborious, inconvenient, and time-consuming [8,13]. More recently, a UHPLC-MS/MS method using a C18 column has been developed for the quantification of β-ODAP, *L*-homoarginine, and asparagine in *Lathyrus sativus* [21]. However, since α-ODAP has not been analyzed, it is possible that both isomers had been quantified simultaneously. Similarly, Gresta et al. (2014) have developed a HPLC-ELS (evaporative light scattering) method for the quantification of both isomers, using a reversed-phase column [14].

Despite the frequent use of reversed phase chromatography for ODAP analysis, hydrophilic interaction chromatography (HILIC) is suitable for analyzing compounds in complex systems that elute near the void (highly polar) in reversed-phase chromatography [23]. HILIC is a variant of normal phase chromatography [8] and typical stationary phases consist of classical bare silica or silica gels modified with many polar functional groups [23]. The predominant retention mechanism in HILIC separation is not always easily predictable [24] and it is commonly accepted that the partitioning of the analyte occurs between two layers [23,24]: the water-deficient mobile phase and water-enriched layer on the surface of the polar stationary phase which is partially immobilized onto the surface of the stationary phase. Nonetheless, other interactions, such as hydrogen-bonding, hydrophobic, and ion-exchange, may also be involved [24]. Precisely defining which mechanism prevails is currently a complex and difficult task [23]. The number of HILIC-compatible columns has increased in the last few years. Generally, stationary phases are silica-based and can often be charged in some pH region [25]. Considering the various types of stationary phases and the poorly understood retention mechanism in HILIC, the choice of an appropriate column for simultaneous analysis of α- and β-ODAP is a challenge [25,26]. Previous HILIC studies have also shown that different selectivities and retention times were obtained using the same mobile phase on different stationary phases [25,26]. Therefore, the possible secondary interactions between the stationary phase and the analytes have to be considered for the column choice [25,26].

HILIC can be conveniently coupled to mass spectrometry (MS), especially in the electrospray ionization (ESI) mode [23]. HPLC-MS/MS is a powerful tandem method for quantification of analytes in complex matrices, providing retention time, mass/charge ratios, and relative abundance (intensity) data [27]. MS/MS detectors are preferred over other chromatographic detectors, due to the better sensitivity, specificity, and wide range applicability [21]. Some authors have employed HILIC columns using MS/MS (tandem mass spectrometry) detectors [8,20] for ODAP analysis. However, these methodologies were not able to distinguish both ODAP isomers.

In this work, a procedure proposed by McKeown (2015) for the selection of the best HILIC-type column was followed [28]. A simple and fast HPLC-ESI-MS/MS method for quantification of both α- and β-ODAP, without sample derivatization, was developed and validated according to the most recent guidelines (European Communities, Food and Drug Administration, European Medicines Agency and Harmonized Tripartite Guideline), and applied to the analysis of 107 grass pea and two red pea (*Lathyrus cicera*) accessions representative of the main grass pea-growing geographical regions in the world, using a neutral HILIC column (chemically bonded diol phase). 

## 2. Results and Discussion

### 2.1. Optimization of the Extraction Procedure

Different ratios of ethanol:water (60:40 [29], 30:70 [16], 70:30 [19]) have been used in the extraction of β-ODAP from grass pea samples). In this work, a higher concentration of ODAP was obtained when samples were extracted with 100% water (higher peaks for both α- and β-ODAP were obtained), and therefore this was the selected solvent for the extraction procedure from grass pea samples. These results are according to Koh et al. (2005), who reported that water was a better solvent than methanol, due to ODAP solubility characteristics [8].

### 2.2. HPLC-MS/MS Method Development

In this work, a HPLC-ESI-MS/MS method was developed, optimized, and validated, for the quantification of both α- and β-ODAP, employing an HILIC column, without sample derivatization. The MS/MS conditions were optimized using a pure β-ODAP standard and the method was tested for its specificity. The positive ionization mode (ESI+) was selected because higher signals were obtained for the precursor ion. Similar findings were obtained by other authors [8,20,21]. Two transitions were used in order to quantify ODAP in samples: MRM (multiple reaction monitoring) 1: 177 > 116 (used for quantification) and MRM2: 177 > 87 (used for qualification). A postulated fragmentation pathway of protonated ODAP was proposed by Koh et al. (2005) [8].

The HPLC method was optimized following the procedure proposed by McKeown (2015) for the selection of the best HILIC column [28]. Three HILIC columns were tested: HILIC-A (acidic character), HILIC-B (basic character), and HILIC-N (bonded neutral character phase), using the mobile phases at the suggested pHs (A: 10 mM ammonium formate in acetonitrile:water 94:6 *v*/*v* and B: 10 mM ammonium formate in acetonitrile:water 50:50 *v*/*v*, at pH 3.0, 4.7, and 6.0) [28].

**Figure 1:**
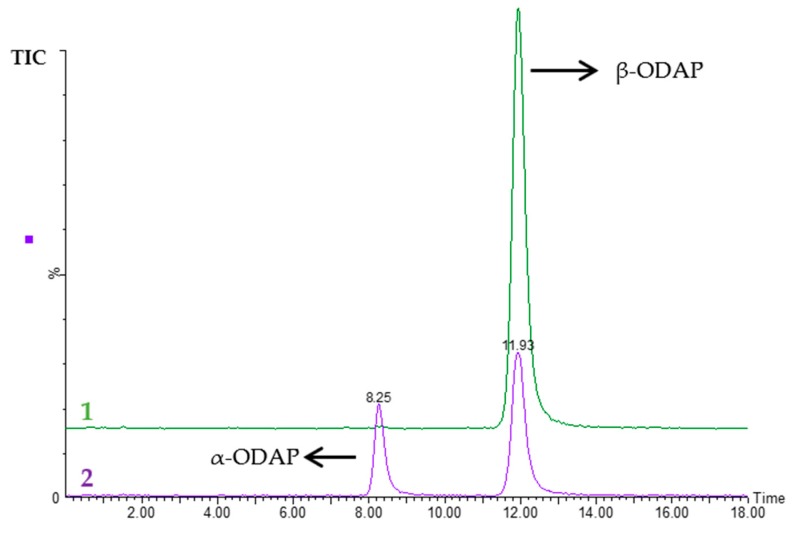
Representative TIC (total ion chromatogram) from MRM (multiple reaction monitoring) chromatograms (*m*/*z* 177 > 116 and *m*/*z* 177 > 87) of (1) a fresh β-*N*-Oxalyl-l-α,β-diaminopropionic acid (β-ODAP) solution at 1 mg L^−1^ and (2) after isomerization to α-ODAP by heating the β-ODAP solution at 120 °C.

When using HILIC-A and HILIC-B, it was not possible to separate α- and β-ODAP. With HILIC-B column, which is characterized by a positive charge on the surface, ODAP was very strongly retained (>120 min, at a flow rate of 0.3 mL min^−1^) when using an isocratic method with 100% eluent B at the three referred pHs. In fact, acidic compounds, such as ODAP, show an increased affinity for positive charged columns, which can sometimes even lead to irreversible adsorption [23]. It was still not possible to separate the two isomers with HILIC-A. When using HILIC-N, however, it was possible to separate α- and β-ODAP, and a very good peak shape was obtained for both compounds. α- and β-ODAP were strongly retained in this column, and the retention times varied according to the mobile phase pH. When using an isocratic method of 100% eluent B at pH 6.0, β-ODAP showed a retention time of 39 min and α-ODAP showed a retention time of 45 min; at pH 4.7, the retention times increased: 42 min for β-ODAP and 50 min for α-ODAP; and at pH 3.0, the retention times decreased, and there was a switch in the elution order of both isomers: α-ODAP showed a retention time of 22 min, while the retention time of β-ODAP was 27 min. The neutral character of HILIC-N suggests there is no change in bonded phase ionization, so it is likely that changes in the analyte ionization states and increasing silanol activity across the pH range explored are responsible for the retention times [28]. Neutral phases demonstrate a high degree of hydrogen bonding with analytes as a principle mode of interaction [23,28]. Changes in eluent pH are also likely to alter the weighting of the hydrogen bonding and other mechanisms with the neutral phase and are the most likely cause of differences in the retention times observed [28]. The pka of β-ODAP is 2.1, which means that, at pH 4.7 and 6.0, the compound has a negative charge, whereas at pH 3.0, some ODAP is unionized, possibly decreasing the hydrogen bonding between the analyte and the column neutral phase, therefore reducing its retention time. In order to further decrease the retention time of ODAP, the eluents pH was decreased until around 1.7, adding 2% HCOOH to eluent A and B, and the acetonitrile concentration of the mobile phase was reduced to 40%, at a flow rate of 0.4 mL min^−1^. It was possible to reduce the retention time of both isomers, without compromising their resolution. Even though it is described that increasing water content (>50%) in the mobile phase results in no longer retention, similarly to that experienced in reversed phase liquid chromatography [24], both ODAP isomers were still retained in the HILIC-N column. In fact, β-ODAP eluted at 12.25 ± 0.21, considering all the control standards and calibration curves analyzed in the present study, in different batches (*n* = 8), for several days. α-ODAP eluted at 8.27 ± 0.01 min (average of six β-ODAP standard solutions after conversion to α-ODAP) (Figure 1).

The retention time of the analytes should be at least twice the retention time corresponding to the void volume of the column [27,30]. The void volume of the column used was approximately 2.5 min, therefore, the retention time of α-ODAP was more than three times the retention time corresponding to the void of the column.

Furthermore, with 2% formic acid, higher signals were obtained for the precursor ion in positive ionization mode (ESI+), causing an increase in the MS/MS signal intensity. This is probably because at this low pH, ODAP is mostly in its nonionized form, being therefore easier to be protonated. At higher pH, more ODAP is in its negatively charged ionized form. Even though the column manufacturer recommends working at two pH units away from an analyte’s pka for robust method developments, this method was selected and validated, due to the shorter run times and the increased sensitivity of ODAP detection.

A similar HPLC-MS/MS method was developed for the quantification of dencichine (β-ODAP) in *Panax* medicinal plant species and in rat plasma, but using 200 mM ammonium formate at pH 3.0 [8]. However, this method, as well as the MS method described by Qian et al. (2012), were used for the quantification of dencichine alone [8,20], which was quantified in the multiple reaction monitoring (MRM) mode using only one transition or product ion (*m*/*z* 177 > 116) [8,20]. The selection of two product ions (MRM1 and MRM2) allows the determination of ion ratio (MRM1/MRM2), which should be within 30% for the confirmation of the compound [27]. MRM1/MRM2 for β-ODAP was 1.55 ± 0.06 (average of all the standards analyzed in different batches), whereas MRM1/MRM2 for α-ODAP was 2.94 ± 0.12 (average of six β-ODAP standard solutions after conversion to α-ODAP), which allows the confirmation of the identification of these compounds in grass pea’s extracts. It is therefore possible that both isomers had been quantified simultaneously in previous works.

**Figure 2:**
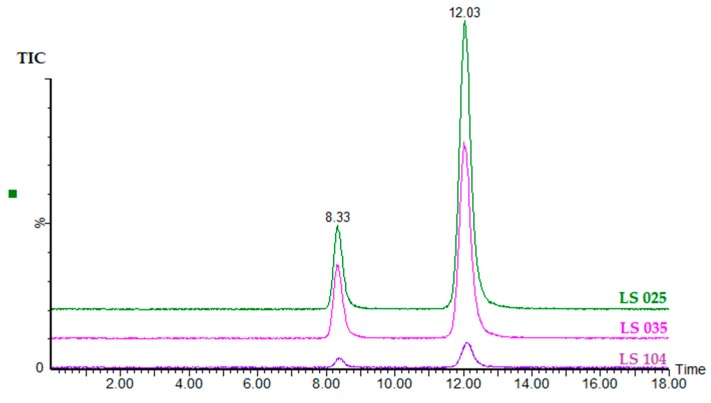
TIC from MRM chromatograms (*m*/*z* 177 > 116 and *m*/*z* 177 > 87) of samples LS 104, LS 035, and LS 025.

After method development, the HILIC-N column was selected, and the mobile phases were 2% HCOOH in acetonitrile (eluent A) and 2% HCOOH in Milli-Q H_2_O (eluent B). An isocratic method consisting of 40% eluent A and 60% eluent B, at a flow rate of 0.40 mL min^−1^, for 18 min, was validated and used for the separation and quantification of α- and β-ODAP in different grass pea accessions. Chromatograms of three samples (LS 104, LS 025, and LS 035), corresponding to different CLs (concentration levels) of β-ODAP, are represented in Figure 2.

### 2.3. Method Validation

#### 2.3.1. Specificity and Selectivity

No peaks were detected when blank assays (40% acetonitrile in water) and other legumes’ extracts (namely faba bean, common bean, chickpea, pea, and lentil extracts) were analyzed using the same HPLC-MS/MS conditions. Carry-over was also assessed, and no peaks were detected after six injections of a β-ODAP standard solution at 3100 ng mL^−1^, corresponding to the ULOQ (upper limit of quantification).

Within sample batches (sample sequences), the retention time of β-ODAP in the extracts corresponded to that of the standard within a tolerance of 0.1 min, which is according to the most recent SANTE/SANCO validation guidelines [27]. The average retention time of β-ODAP in all samples (six batches) was 12.30 ± 0.25 min, and the relative standard deviation (RSD) was 2.3%, whereas the retention time of α-ODAP was 8.51 ± 0.17 min, and the RSD was 2.0%.

MRM1/MRM2 ratio was also determined for both isomers in all samples analyzed, and its deviation was always lower than 9%, which is below the 30% described in the most recent SANTE/SANCO validation guidelines [27]. Considering all the samples studied, the RSD of the MRM1/MRM2 ratio for β-ODAP was 1.3%, and 2.7% for its α-isomer. Hence, the method was considered specific for α- and β-ODAP analysis.

#### 2.3.2. Limit of Detection (LOD) and Lower Limit of Quantification (LLOQ)

**Figure 3:**
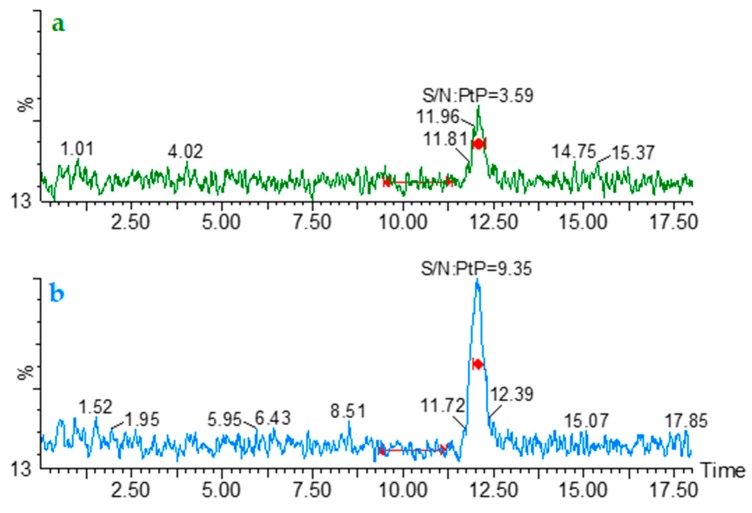
Representative MRM1 chromatograms (*m*/*z* 177 > 116) of (**a**) β-ODAP solution at 10 ng L^−1^ (limit of detection—LOD) and (**b**) β-ODAP solution at 25 ng L^−1^ (lower limit of quantification—LLOQ). PtP—Peak to Peak.

The limit of detection is the lowest amount of analyte in a sample which can be detected but not necessarily quantified as an exact value [31]. A signal-to-noise ratio of 2:1 or 3:1 is generally considered acceptable for estimating the detection limit [31]. In this work, a signal-to-noise ratio (S/N) of 3:1 was obtained when six independent β-ODAP solutions at 10 ng mL^−1^ were analyzed using the analytical conditions of the implemented method (Figure 3).

The lower limit of quantification (LLOQ) of an individual analytical procedure is the lowest amount of analyte in a sample which can be quantitatively determined with suitable precision and accuracy [32,33], with typical S/N ratio of 5:1 to 10:1 [31,32,33]. A S/N around 9:1 was obtained when six β-ODAP solutions at 25 ng mL^−1^ were analyzed using the analytical conditions of the implemented method (Figure 3).

The limit of detection (LOD) and LLOQ obtained were lower than those reported by Koh et al. (2005) for the analysis of dencichine in Panax medicinal plant species by HPLC-MS/MS, using an HILIC column (LOD of 300 ng mL^−1^ and LLOQ of 1500 ng mL^−1^) [8,20], and similar to the values reported by Qian et al. (2012) for the analysis of dencichine in plasma by HPLC-MS/MS, using a C18 column (LLOQ of 20 ng mL^−1^) [20]. However, in the latter method, the compound seems to be eluting in the void volume, and did not distinguish β- from α-ODAP. Higher LOD and LLOQ were obtained for the analyses of β-ODAP by HPLC without derivatization (5.6 and 16.9 μg mL^−1^) [13], by GC-MS after derivatization (0.5 and 2.0 μg mL^−1^) [9], by HPLC after derivatization (26.4 and 88 ng mL^−1^) [18], by UHPLC-MS/MS (1.51 μg mL^−1^) [21], and by capillary electrophoresis (2.0 and 6.6 μg mL^−1^) [16].

The low quantification limit obtained in our work may be useful for future experiments which aim to quantify trace amounts of α- or β-ODAP, such as pharmacokinetic assays in biological samples, or for the possible detection of α-ODAP in *Panax* medicinal plant species, which present lower amounts of ODAP (3 μg g^−1^) [8].

#### 2.3.3. Linearity and Linear Range

The linearity of the method for β-ODAP was verified, and the determination coefficient (r^2^) was calculated using the least-square regression method. A linear calibration curve was obtained over the quantification range, for each batch of analysis (including validation assays): y = (42.85 ± 1.90) x – 284.27 ± 120.52, with determination coefficients higher than 0.9998. The back calculated concentrations of all calibration standards (*n* = 9) were within ±10% of the nominal value, except the LLOQ which was within ±14%. Therefore, all calibration curves met the validation criteria specified in different guidelines: back calculated concentrations of at least 75% of a minimum of six calibration standards within ±15% of the nominal value and ±20% of the nominal value for the LLOQ [32,33].

#### 2.3.4. Precision and Accuracy

The precision was evaluated at three different levels: injection repeatability, experimental repeatability, and inter-day precision. Results are reported in Table 1. The relative standard deviation of each concentration level (CL) analyzed was below 6.5%, lower than the reference values of 15% and 20% for the LLOQ [32,33]. Other developed methodologies for ODAP quantification by HPLC-MS/MS reported a precision below 6.7% [8,20]. Injection repeatability in three real samples (LS 104, LS 035, and LS 025), corresponding to different CLs of α- and β-ODAP, was below 1.7% for β-ODAP and 5.4% for α-ODAP.

The developed method presented a remarkable accuracy, within ±3.5% of the nominal values, below the reference values of ±15% and ±20% at LLOQ described in different guidelines [31,32,33]. The method was therefore considered precise and accurate for the detection of β-ODAP.

#### 2.3.5. Matrix Effect

Matrix effect (ion suppression or enhancement) may be caused by the presence of unintended analytes or other interfering substances in the sample [33]. The slope value of six calibration curves prepared in matrices (grass pea extracts) were compared to the slope of calibration curve prepared in solvent (calibration curve of the respective sample batch). The matrix effect was considered negligible (Table 2), since the highest deviation of the slopes obtained in the matrix was 14%, and the average matrix factor was 95% ± 6.49%. Therefore, it was decided to prepare the calibration curve for β-ODAP quantification in solvent (acetonitrile:water 40:60).

#### 2.3.6. Dilution Integrity

Dilution (1:50) of samples in acetonitrile: water (40:60) did not affect the method accuracy (Table 3), and all the spiked samples were within ±13% of their nominal concentrations.

#### 2.3.7. Method Recovery

Recovery refers to the extraction efficiency of an analytical process [30,33]. Recoveries were between 91% ± 4% and 104% ± 9% (Table 4). Other methods for ODAP quantification report ODAP recoveries of 85%–106.09% [8,13,20]. 

#### 2.3.8. Stability Assays

A study on the stability of β-ODAP is mandatory, once a spontaneous isomerization to α-ODAP [2,5,14] and a hydrolysis to DAP [8,11,13,15,16] may occur. It is known that these reactions are strongly influenced by pH and heating and, at equilibrium conditions, a mixture of 40% α- and 60% β-ODAP is developed [5,11,14]. In order to verify if isomerization and hydrolysis took place during the experimental procedures used in this study, a stability study was performed with pure β-ODAP standards, as well as with grass pea extracts. According to criteria described in different guidelines, a maximum accuracy of 15% was considered for measuring β-ODAP stability [32,33].

**Figure 4:**
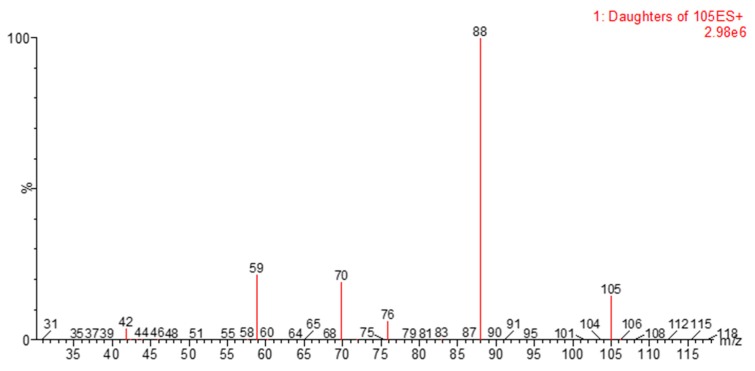
Collision-induced dissociation (CID) spectra of diaminopropionic acid (DAP).

The possibility of hydrolysis of ODAP to DAP in grass pea extracts was investigated by HPLC-MS/MS, using the MRM transitions *m*/*z* 105 > 88 and 105 > 59, and comparing to a pure DAP standard. The MS/MS instrumental parameters used were similar to those used for ODAP. A similar experiment was done by Koh et al. (2005), but using different transitions: *m*/*z* 105 > 88 and 105 > 70 [8]. The fragment *m*/*z* 70 was also found in DAP standard, but its intensity was lower than *m*/*z* 59 (Figure 4).

β-ODAP solutions were stable (stock solution of 40 mg L^−1^ in water and working solutions), at all conditions tested: room (23 °C), autosampler (10 °C), and freezer (−20 °C) temperatures (Table 5). The isomerization of β- to α-ODAP only started to occur after one week at room temperature. Only a small amount of β-ODAP, 4.5% ± 0.3%, was converted to its α isomer when using acetonitrile:water (40:60) as solvent. In water, β-ODAP stability was lower, and 7.3% ± 0.7% isomerized to α-ODAP. After two months in water, at room temperature, the content of α-ODAP was around 27.1% ± 0.31% of the total ODAP content. These findings are according to those reported by Gresta et al. (2014), and show that the isomerization of β- to α-ODAP is very slow and did not occur during the experimental period of the current study [14]. Stock β-ODAP solutions remained stable after three complete freeze-thaw cycles.

A complete stability study was also carried out with sample extracts, to ensure that every step taken during sample preparation and sample analysis, as well as the storage conditions used, do not affect the concentration of α- and β-ODAP in grass pea extracts [32].

β- and α-ODAP showed a very good stability in the matrix, before acetonitrile:water (40:60) dilution, in all the conditions tested: room temperature (23 °C), fridge (4 °C), and freezer (−20 °C) (Table 6). Grass pea extracts (before dilution) remained stable after three complete freeze-thaw cycles. On the other hand, ODAP content in the extract corresponding to the low CL (LS 104) showed some differences when stored in acetonitrile:water (40:60), i.e., after dilution, after freezing, and after one week in the fridge (Table 6). Diluted extracts presented a good stability in the autosampler (10 °C), after one week, and at room temperature, after 24 h. Therefore, grass pea extracts should be stored at −20 °C in water, before acetonitrile:water (40:60) dilution, diluted before HPLC-MS/MS analysis, and kept at 10 °C in the autosampler, for a maximum of one week. The stability assays confirm that α- and β-ODAP contents detected are the real amounts of the two isomers in the grass pea accessions studied.

### 2.4. Determination of ODAP Content in Lathyrus sativus Accessions

The LC-MS/MS method described in the present work was considered adequate for the intended purposes and was used to analyze α- and β-ODAP contents in 107 grass pea and two red pea accessions. The quality controls (QCs) used to assess the inter-day precision and accuracy of the assay met the calibration acceptance criteria, and all the QCs were ±14% of the nominal values, which therefore proved the integrity and validity of the results [33].

The average β- and α-ODAP contents in grass pea samples (*n* = 107) were 3.49 ± 0.84 mg g^−1^ and 1.09 ± 0.27 mg g^−1^ (expressed as β-ODAP equivalents), respectively, corresponding to a total ODAP content of around 4.58 ± 1.04 mg g^−1^.

**Figure 5:**
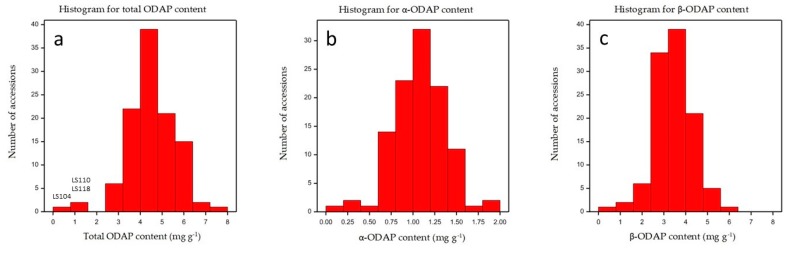
Frequency distribution of (**a**) total ODAP (α and β), (**b**) α-ODAP, and (**c**) β-ODAP contents, expressed as mg g^−1^ sample in a collection of 107 grass pea plus two red pea accessions. Total ODAP content of the low ODAP grass pea accession (LS 104) and of the two red pea accessions (LS 110 and LS 118) are indicated.

**Figure 6:**
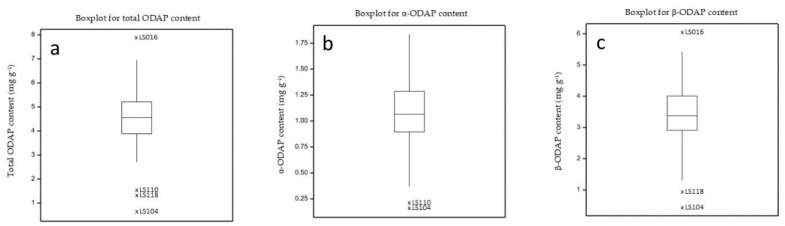
Boxplot distribution of (**a**) total ODAP (α and β), (**b**) α-ODAP, and (**c**) β-ODAP contents, expressed as mg g^−1^ sample in a collection of 107 grass pea plus two red pea accessions.

The content of β-ODAP in *Lathyrus cicera* (red pea) is usually reported as being lower than in *L. sativus* [18,29]. The two red pea accessions analyzed (LS110 and LS118) showed β-ODAP contents of 1.32 ± 0.06 and 0.94 ± 0.03 mg g^−1^, which are according to the values described in the literature (0.79–3.02 mg g^−1^) [18,29] and lower than the *L. sativus* average values (3.49 mg g^−1^). However, the grass pea sample LS 104 presented a lower β-ODAP content (0.45 ± 0.02 mg g^−1^). This was not surprising, since this accession is a *L. sativus* line derived from the accession LS87124 developed by Clayton Campbell (Agriculture and Agrifood Canada, Morden) and known for its lower ODAP content (F. Lambein, personal communication) (Figure 5 and Figure 6).

An indicative β-ODAP content lower than 1.5 mg g^−1^ in seeds of *L. sativus* has been proposed as safe for human consumption [34]. β-ODAP content on the remaining 106 grass pea samples ranged from 1.94 ± 0.11 to 6.04 ± 0.45 (Table S1). Other studies using grass pea accessions from different origins, have detected β-ODAP concentrations from 0.9 to 10.2 mg g^−1^ [14,16,17,18,19,29]. LS 104 also showed the lowest α-ODAP content (0.16 ± 0.005 mg g^−1^) of all grass pea samples analyzed in the present study, and the level of this isomer in the other samples ranged from 0.56 ± 0.02 to 1.84 ± 0.14 mg g^−1^. We can conclude that the high genetic diversity among the grass pea accessions under study was reflected by a high diversity in ODAP isomers content.

ODAP content in *Lathyrus* species can be affected by a wide range of environmental conditions [35], which include water stress [19], soil pH, soil potassium level, and sunshine hours (which cause an increase in the concentration of the toxin) [36] and salinity (which decreases β-ODAP concentration) [2,19,35]. Different results have been obtained for environments with higher altitudes [14,36]. On the other hand, it has been established that a low β-ODAP genotype maintained the lowest β-ODAP concentration at all levels of water availability [19]. In the present study, all grass pea samples were exposed to the same edapho-climatic conditions, therefore, the differences in α- and β-ODAP content among samples should be a consequence of genetic factors of the different accessions. The detected interesting sources of low β-ODAP may be used in future breeding programs as pre-breeding material to improve grass pea quality. Nevertheless, further trials in different environments will be fundamental to estimate the environmental effects and the genotype × environmental interaction on the β-ODAP content for the potential development of broad or locally adapted higher quality varieties.

**Figure 7:**
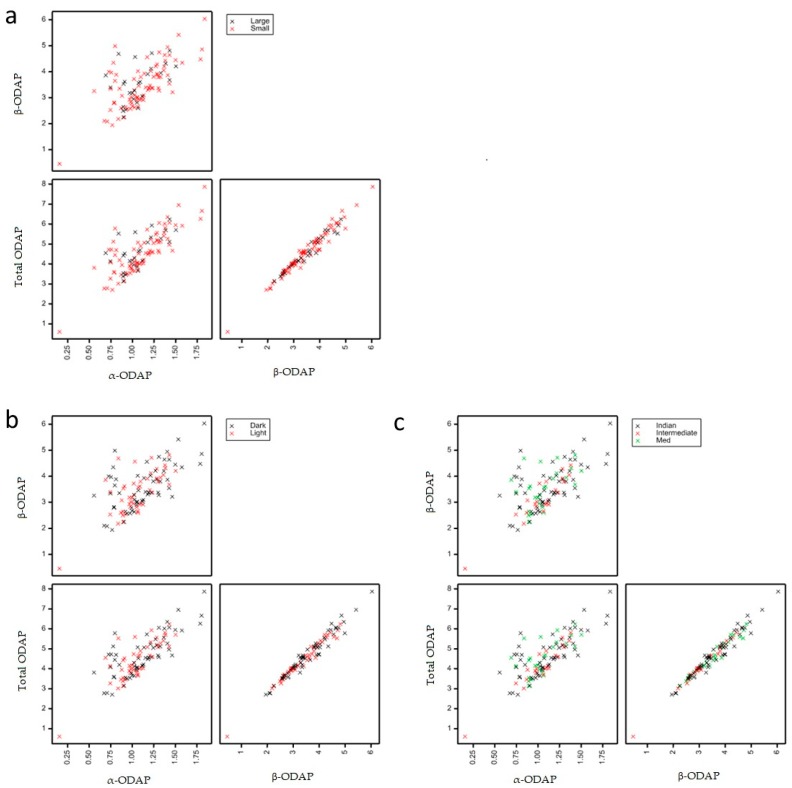
Scatter plot matrix of (**a**) total ODAP (α and β), (**b**) α-ODAP, and (**c**) β-ODAP contents, expressed as mg g^−1^ of sample in a collection of 107 grass pea accessions, highlighting (**a**) seed size, (**b**) seed color, and (**c**) varietal group.

It has been described that the natural abundance of α-ODAP is approximately 5% of the total ODAP in seeds of *L. sativus* [5,17]. However, in a recent study published by Megías et al. (2015), it was found that the content of β-ODAP was about four to seven times higher than that of α-ODAP in *L. cicera*, and around eight times higher in *L. sativus* [18], meaning that more than 5% of total ODAP was in its α form and that the natural abundance of α-ODAP may vary. Although in the current study α-ODAP was expressed as β-ODAP equivalents, it was possible to confirm these results, and differences in the natural abundance of α-ODAP in grass pea seeds were detected. In fact, β-ODAP content was two to seven times higher than that of α-ODAP, and β-ODAP represented around 69% ± 0.2% to 86% ± 0.2% of the total ODAP content. These results were also similar to the results obtained by Gresta et al. (2014), who have also quantified α-ODAP as β-ODAP equivalents (the β-ODAP content was four to seven times higher than that of α-ODAP in the 10 Sicilian accessions of grass pea) [14]. The relative standard deviations obtained for the total of β-ODAP of sample triplicates were always below 1.1%. Indeed, a moderate correlation (Pearson correlation coefficient: 0.6528) was found between the α- and β-ODAP contents (Figure 7).

Additionally, no significant differences in the β-ODAP contents were found between grass pea seed size groups (large and small, *p*-value = 0.712), seed color groups (light and dark, *p*-value = 0.196) or varietal groups (Mediterranean, Indian, or intermediate types, *p*-value = 0.086) contrary to the broadly described association of large seed Mediterranean varietal types and lower ODAP content [37]. This lack of differentiation highlights the possibility to select identical β-ODAP content accessions within different varietal groups.

## 3. Materials and Methods

### 3.1. Chemicals and Materials

For extract preparation and HPLC-MS/MS analysis, Milli-Q water (18.2 MΩ.cm resistivity) was obtained from a Millipore-Direct Q3 UV system (Millipore^®^, Burlington, MA, USA); formic acid ≥ 95% was from Sigma-Aldrich^®^, St Louis, MO, USA; and acetonitrile HPLC Plus Gradient was from Carlo Erba^®^, Val de Reuil, France. β-ODAP standard was obtained from Lathyrus Tecnhologies, Hyderabad, India.

Since the isomerization of β- to α-ODAP increases with increase in temperature [38], six solutions of β-ODAP in water, at 40 mg L^−1^, were heated at 120 °C for 120 min. These solutions were used for HPLC-MS/MS method development.

### 3.2. Samples

A collection of 107 grass pea accessions representative of the main grass pea-growing geographical regions of the world plus two *L. cicera* accessions, kindly provided by Prof. Fernand Lambein (IPBO, Ghent, Belgium), USDA-ARS (Pullman, WA, USA), INIA-CRF (Madrid, Spain), IAS-CSIC (Córdoba, Spain), ICARDA, HAO-DEMETER (Thermi, Greece), and IFVC (Novi Sad, Serbia) germplasm collections, were analyzed for their α-ODAP and β-ODAP contents under the scope of the European FP7 project LEGATO-LEGumes for the Agriculture of TOmorrow. Accessions were grouped in big (>20 g/100 seeds weight) or small seed size, light or dark seed color, and as a combination of both traits into varietal groups [39]: Mediterranean type (big light seeds), Indian type (small dark seeds), and all the other size and color combinations in an Intermediate group (Table S2).

All the accessions were multiplied under the same edapho-climatic conditions at the IAS-CSIC experimental farm (Córdoba, Spain) and kindly provided by Prof. Diego Rubiales. Dry mature seeds of grass pea were milled (Falling n° 3100–Perten, Sweeden) to a particle size of 0.8 mm and flour stored at −20 °C until analysis.

### 3.3. Optimization of the Extraction Procedure

In order to achieve a higher ODAP extraction efficiency, water and different ethanol:water solvent ratios (60:40, 30:70, 70:30) described in the literature for ODAP extraction [8,14,16,19,29] were tested, using commercial grass pea samples, based on methods previously described [8,14,16,22,38]. Water was selected as the best extraction solvent. Briefly, samples were accurately weighed (48 mg) and ODAP (α and β) was extracted with 2 mL of solvent, vortexed for 90 s, and extracted for 2 h, in ice, using a Selecta^®^ Rotabit orbital shaker and centrifuged (20 min, 10,000 rpm). Extracts were kept at −20 °C until analysis, for a maximum of two months. Before HPLC-MS/MS analysis, extracts were diluted (1:50) in acetonitrile:water 40:60, in order to match the mobile phase, and filtered through a 0.20 μm PTFE syringe filter (Chromafil^®^ Macherey-Nagel, Germany). Extracts were prepared in triplicate.

### 3.4. HPLC-MS/MS Method Development

The HPLC-MS/MS analyses were performed on a Waters Alliance HPLC system (Waters^®^, 2695 separation module, Dublin, Ireland) comprising a quaternary pump, an on-line solvent degasser, autosampler, and column oven. The HPLC method was optimized following the procedure proposed by McKeown (2015) for the selection of the best HILIC column: HILIC-A, HILIC-B, and HILIC-N (100 Å, 5 µm 3.0 × 150 mm), ACE^®^, Scotland, using the mobile phases A: 10 mM ammonium formate, pH 3.0, 4.7 or 6.0 in acetonitrile:water (94:6 *v*/*v*) and B: 10 mM ammonium formate, pH 3.0, 4.7, or 6.0 in acetonitrile:water (50:50 *v*/*v*) [28].

The MS/MS conditions were optimized using a pure β-ODAP standard. Tandem mass spectrometry (MS/MS) detection was performed on a Micromass^®^ Quattro Micro triple quadrupole (Waters^®^, Ireland) using an ESI source operating at 140 °C, applying a capillary voltage of 2.7 kV and a cone voltage of 10 V, in positive ion mode. MassLynx software (version 4.1) was used to control the system, for data acquisition and processing. High purity nitrogen (N_2_) was used both as drying gas and as a nebulizing gas. Ultra-high purity argon (Ar) was used as collision gas. Collision energies were optimized using a β-ODAP standard solution and was set at 10 eV. The analysis was performed in MRM mode in order to achieve a higher selectivity and sensitivity. 

After method optimization, validation and application to real samples were performed in a HILIC-N (bonded neutral character phase), column at 25 °C, using an injection volume of 10 µL. The mobile phase consisted of an isocratic method of 40% of 2% HCOOH in acetonitrile (eluent A) and 60% of 2% HCOOH in Milli-Q H_2_O (eluent B) at a flow rate of 0.40 mL min^−1^, for 18 min. Autosampler temperature was set at 10 °C.

### 3.5. Method Validation

Validation was carried out by determination of specificity, limit of detection (LOD), and quantification (LLOQ), linearity and linear range, precision, accuracy, matrix effect, dilution integrity, recovery, and stability assays.

#### 3.5.1. Specificity

Since ODAP is an endogenous compound of grass pea, it was not possible to determine the specificity in grass pea extracts. Extracts of other legumes were prepared and analyzed, namely faba bean (*Vicia faba*) (*n* = 1), common bean (*Phaseolus vulgaris*) (*n* = 1), chickpea (*Cicer arietinum*) (*n* = 1), pea (*Pisum sativum*) (*n* = 2), and lentils (*Lens culinaris*) (*n* = 2), and analyzed as blank assays.

A standard solution of β-ODAP at 100 mg L^−1^ was infused into the mass spectrometer in order to determine the two product ions with the highest signals. These transitions were used as the quantification transition (MRM1) and the confirmation transition (MRM2), in order to evaluate the method specificity. The same transitions were used for the quantification of α-ODAP. MRM1/MRM2 signal transition ratio was determined for α- and β-ODAP and compared with the values obtained for the sample extracts. The retention time of both isomers in solution and samples was also compared. Carry-over was addressed by injecting blank samples after six injections of a high concentration calibration standard of β-ODAP at 3100 ng mL^−1^, corresponding to the upper limit of quantification (ULOQ).

#### 3.5.2. Limit of Detection (LOD) and Lower Limit of Quantification (LLOQ)

A β-ODAP solution of 40 mg L^−1^ was prepared in water and diluted in acetonitrile:water (40:60), until a signal-to-noise ratio of 3:1 (LOD) and 10:1 (LLOQ). The LOD and LLOQ values were confirmed by the analysis of six solutions at these concentrations, prepared from six independent stock solutions (around 40 mg L^−1^).

#### 3.5.3. Linearity and Linear Range

The linearity study was performed using nine calibration standards, in order to cover the quantification of α- and β-ODAP simultaneously, with the concentrations of 25 ng mL^−1^ (LLOD), 75, 100, 300, 700, 1300, 1900, 2500, and 3100 ng mL^−1^ (ULOQ). All the calibration standards in the different batches (*n* = 8) were prepared from fresh β-ODAP standard solutions of around 40 mg L^−1^ in water, and diluted in acetonitrile:water (40:60), in order to match the mobile phase constitution. The determination coefficient (r^2^) was calculated. Since no commercial standard is available, α-ODAP was quantified using the β-ODAP calibration curve and expressed as β-ODAP equivalents.

#### 3.5.4. Precision and Accuracy

Accuracy (intra-day and inter-day accuracy) and precision (experimental precision, injection repeatability, and inter-day precision) were determined at the LLOQ (25 ng mL^−1^), low (75 ng mL^−1^), mid (1300 ng mL^−1^), and high (2500 ng mL^−1^) β-ODAP concentration levels (CLs).

For the determination of intra-day accuracy and experimental repeatability, six β-ODAP solutions at approximately 40 mg L^−1^ were prepared in water, and diluted to the LLOQ, low CL, mid CL and high CL in acetonitrile:water (40:60), and their concentrations were calculated against a calibration curve. In order to determine the injection repeatability, one solution at the LLOQ, low CL, mid CL, and high CL was injected 10 times in the equipment.

To evaluate the inter-day accuracy and precision, two β-ODAP solutions were prepared for each sample batch (sequence of analysis) and diluted to the same CLs described above (LLOQ, low, mid, and high). All the quality control (QC) standards analyzed in all sample batches (*n* = 8) were used for the determination of inter-day accuracy and precision. Their concentrations were determined against the calibration curve of the respective sample batch, and compared with their nominal values. Accuracy was reported as percent of the nominal value [32,33], and precision was expressed as relative standard deviation (RSD) [30,32,33].

Injection repeatability was also evaluated analyzing three real samples (LS 104, LS 035, and LS 025), corresponding to different CLs of α- and β-ODAP, and results were expressed as relative standard deviation (%).

#### 3.5.5. Matrix Effect

To address matrix effects, ion suppression, or enhancement, six calibration curves were prepared in six grass pea’s extracts. Since β-ODAP is an endogenous compound of grass pea, slopes of the curves in pure solvent (acetonitrile:water (40:60)) and in sample matrix were compared, and the matrix factor (MF) was quantified as the ratio between the slope of the matrix calibration curve and pure solvent [40].

Extracts of six samples (LS 043, LS 067, LS 076, LS 104, LS 114, and LS 118) were selected to study the matrix effect, since they have different endogenous CLs of β-ODAP. Water extracts were diluted (1:50) in acetonitrile:water (40:60), and 180 μL were spiked with 20 μL of different CLs (0.25 to 31 μg mL^−1^) of β-ODAP standards in solvent (acetonitrile:water (40:60)), to obtain spiked concentrations of 25, 75, 100, 300, 700, 1300, 1900, 2500, and 3100 ng mL^−1^ of β-ODAP in the samples. The background peak areas (endogenous peak areas) of β-ODAP in the matrices were determined adding 20 μL of solvent to 180 μL of each extract. In order for this method to be reproducible, only the CLs which gave an increase of >20% in the matrix peak areas (endogenous β-ODAP content of the samples mentioned above) after spiking with β-ODAP were considered for the calibration curve in the matrices [41].

#### 3.5.6. Dilution Integrity

Six grass pea extracts randomly chosen (LS 043, LS 067, LS 076, LS 103, LS 104, and LS 114) were spiked with 10 mg L^−1^ of β-ODAP in water, before dilution. Samples were diluted 1:50 in acetonitrile:water (40:60), analyzed in the HPLC-MS/MS, and compared to non-spiked extracts. The accuracy was determined for each sample. Results were expressed as %.

#### 3.5.7. Method Recovery

To address method recoveries, 48 mg of four different samples (LS 059, LS 075, LS 104, and LS 114) were extracted with 2 mL of β-ODAP aqueous solutions of 3.75, 65, and 125 mg L^−1^. Thus, after extracts dilution (1:50), β-ODAP concentrations corresponded to 75 ng mL^−1^ (low CL), 1300 ng mL^−1^ (mid CL), and 2500 ng mL^−1^ (high CL), respectively. Peak areas of the extracted samples were compared to the peak areas of samples spiked with the analyte post-extraction. Recovery was reported as a percentage of the known amount of the analyte carried through the sample extraction and processing steps of the method [30,33].

#### 3.5.8. Stability Assays

The stability of β-ODAP in solution (stock solution and working solutions) was evaluated. Two fresh β-ODAP stock solutions were prepared in water (40 mg L^−1^), diluted to a high, mid, and low CL (2500, 1300, and 75 ng mL^−1^) in acetonitrile:water (40:60) (working solutions) and analyzed by HPLC-MS/MS. The stability of the β-ODAP stock solutions was evaluated at −20 °C, up to three months. Autosampler stability of β-ODAP working solutions in acetonitrile:water (40:60) was also evaluated, up to one week. The stability of stock solutions (40 mg L^−1^) and working solutions of β-ODAP was also evaluated at room temperature (23 °C), up to two months. Freeze-thaw stability of the two β-ODAP stock solutions was also verified after three cycles: stock solutions were stored and frozen in the freezer at −20 °C for at least 24 h, and thereafter thawed at room temperature. After complete thawing, β-ODAP stock solutions were refrozen again, in the same conditions. Control standards were analyzed against fresh calibrations curves prepared in solvent, and the obtained concentrations compared to the nominal concentrations.

The stability of α- and β-ODAP in the sample matrix (grass pea extracts in water and after 1:50 dilution in acetonitrile:water (40:60)) was evaluated in the freezer (−20 °C), up to three months; fridge (4 °C), up to one week; and room temperature (23 °C), up to two months. Freeze-thaw stability (three cycles) was also evaluated. The autosampler stability was verified in the diluted extracts, up to one week. Three samples were chosen for the stability assay, corresponding to a low (LS 104), mid (LS 035), and high (LS 025) CLs of ODAP, and were analyzed as triplicates. Samples were analyzed against fresh calibrations curves prepared in solvent, and the obtained concentrations compared to the fresh extracts’ initial concentrations.

### 3.6. Application of the Method

ODAP was extracted from 107 grass pea and two red pea accessions, and its content was analyzed by HPLC-MS/MS following the developed methodology, using freshly prepared extracts. The HPLC-MS/MS analysis was performed for all the extracted samples (triplicates of extraction), and a calibration curve was analyzed for each batch of analysis (*n* = 8), to ensure accuracy. Blanks (acetonitrile:water (40:60)) and duplicates of QCs standards at three CLs (75, 1300, and 2500 ng mL^−1^ of β-ODAP), were also run between every 20 injections to verify the instrument response. Summary statistics and correlations were calculated (GenStat, 19th Ed.).

## 4. Conclusions

In this study, a HPLC-MS/MS method for the quantification of α- and β-ODAP in grass pea samples was developed, optimized, and validated. The method was validated according to the most recent guidelines (European Communities, Food and Drug Administration, European Medicines Agency and Harmonised Tripartite Guideline), and applied to the analysis of 107 grass pea and two red pea accessions. The developed methodology allows a simple and fast quantification of the neurotoxin β-ODAP along with its non-toxic α isomer, in several grass pea samples, since it does not involve sample derivatization steps. The method is able to detect low amounts of both compounds, with a good precision and accuracy, which may be useful in future analysis of Panax medicinal plant species or bioavailability studies. In the samples analyzed in the present study, β-ODAP content ranged from 0.45 ± 0.02 to 6.04 ± 0.45 mg g^−1^, and β-ODAP content represented around 69% ± 0.2% to 86% ± 0.2% of the total ODAP content. Results showed the presence of contrasting accessions in what concerns the α- and β-ODAP contents. The moderate correlation found between α- and β-ODAP contents (0.65) reinforces the importance of the independent quantification of both ODAP isomers.

## Figures and Tables

**Table 1:** Intra-day and inter-day precision and accuracy of β-ODAP. Results are expressed as %.

Concentration (ng mL^−1^)	Intra-Day Precision		Inter-Day Precision	Intra-Day Accuracy	Inter-Day Accuracy
	Injection Repeatability	Experimental Repeatability			
25	5.3	4.5	4.4	0.7	1.1
75	3.3	5.8	6.5	3.5	0.4
1300	0.6	2.0	5.5	2.2	1.3
2500	1.0	0.6	5.1	0.9	0.1

**Table 2:** Matrix effect on β-ODAP determination in different samples. MF—Matrix Factor.

	Solvent	LS 043	LS 067	LS 076	LS 104	LS 114	LS 118	Average (Matrix)
Slope	45.32	40.26	41.19	45.20	39.16	46.79	44.40	43.16 ± 2.94
MF (%)	100	86	103	98	100	95	89	95 ± 6.49

**Table 3:** β-ODAP dilution integrity (accuracy, %) in different grass pea samples.

LS 103	LS 104	LS 114	LS 076	LS 067	LS 043
3	13	12	6	3	2

**Table 4:** Method recovery of β-ODAP extraction in different samples, at 75 (low concentration level (CL)), 1300 (mid CL), and 2500 (high CL) ng mL^−1^. Results expressed as %.

Concentration (ng mL^−1^)	LS 059	LS 075	LS 104	LS 114	Average
75	94	107	99	114	104 ± 9
1300	94	100	86	107	97 ± 9
2500	90	92	86	95	91 ± 4

**Table 5:** β-ODAP stability determined as accuracy and expressed as %, for β-ODAP stock solution (S) and working solutions (W). TC—Theoretical Concentration, expressed as ng mL^−1^. Values in bold: accuracy > 15%.

Theoretical Concentration (ng mL^−1^)			75	1300	2500
Freshly prepared solutions		Solution 1	0	1	1
		Solution 2	7	1	3
Room temperature (23 °C)	1 week	W 1	10	1	1
		W 2	0	3	5
	1 week	S 1	3	10	9
		S 2	**41**	9	9
	2 months	S 1	**16**	**21**	**22**
		S 2	**19**	**20**	**19**
Autosampler (10 °C)	3 days	W 1	6	8	7
		W 2	2	6	5
	1 week	W 1	11	1	2
		W 2	3	1	1
Freeze-thaw (−20 °C)	Cycle 1	S 1	3	6	5
		S 2	5	5	6
	Cycle 2	S 1	10	11	13
		S 2	8	9	11
	Cycle 3	S 1	3	3	3
		S 2	3	5	7
Freezer (−20 °C)	3 months	S 1	8	5	4
		S 2	6	4	4

**Table 6:** β-ODAP stability determined as accuracy and expressed as % for sample extracts before dilution (water extracts, W) and after dilution with acetonitrile:water (40:60) (A:W). Values in bold: accuracy > 15%.

Samples			LS 104		LS 035		LS 025	
α and β-ODAP			α	β	α	β	α	β
Room temperature (23 °C)	24 h	W 1	2	2	1	5	2	0
		W 2	2	1	5	11	6	6
		W 3	6	1	8	12	4	10
		A:W 1	3	2	0	2	2	3
		A:W 2	2	0	3	8	6	8
		A:W 3	0	1	5	10	8	12
	1 week	W 1	10	**66**	7	3	**97**	**22**
		W 2	**23**	6	4	4	**80**	**18**
		W 3	**22**	5	5	2	**48**	**22**
		A:W 1	**21**	10	**16**	7	**25**	**17**
		A:W 2	**25**	9	14	1	**21**	9
		A:W 3	**25**	11	15	10	**35**	**27**
Autosampler (10 °C)	1 week	A:W 1	1	4	5	8	2	2
		A:W 2	3	5	8	14	4	7
		A:W 3	7	2	8	12	4	5
Fridge (4°C)	1 week	W 1	3	1	6	8	2	1
		W 2	6	1	7	12	7	6
		W 3	4	1	9	13	7	12
		A:W 1	**20**	**19**	4	5	13	11
		A:W 2	**22**	**16**	2	3	7	4
		A:W 3	**37**	**30**	1	1	2	2
Freeze-thaw (−20 °C)	Cycle 1	W 1	2	3	1	1	1	1
		W 2	0	2	2	8	5	6
		W 3	6	3	5	9	9	11
		A:W 1	6	12	1	2	3	3
		A:W 2	**16**	9	0	5	6	5
		A:W 3	**17**	7	0	2	1	2
	Cycle 2	W 1	14	5	3	0	5	5
		W 2	12	2	0	6	1	0
		W 3	11	8	3	7	2	5
		A:W 1	15	9	6	3	5	2
		A:W 2	13	14	3	4	8	5
		A:W 3	**28**	**21**	3	3	6	0
	Cycle 3	W 1	7	2	1	0	11	10
		W 2	12	5	0	6	10	9
		W 3	13	11	6	3	7	4
		A:W 1	11	5	3	4	1	3
		A:W 2	10	2	6	11	1	1
		A:W 3	**33**	**18**	5	8	2	6
Freezer (−20 °C)	3 months	W 1	7	4	1	3	4	3
		W 2	8	7	0	7	1	2
		W 3	13	6	2	6	3	6

## Supplementary Materials

The following are available online, Table S1: α and β-ODAP content (mg g^−1^) in *Lathyrus sativus* accessions, Table S2: Germplasm bank, geographical origin, and seed size, color, and varietal group characterization.
