# Supplementary material for: An Improved HILIC HPLC-MS/MS Method for the Determination of β-ODAP and Its α Isomer in Lathyrus sativus
Source: Molecules. 2019 Aug 22;24(17):3043. doi: 10.3390/molecules24173043 (PMC6749377; doi:10.3390/molecules24173043)
Supplement: Supplementary file 1 [file molecules-24-03043-s001.pdf]

Table S1. α- and β-ODAP contents (mg g^{-1}) and % β-ODAP of *Lathyrus sativus* and *Lathyrus cicera* (*) samples.

Sample	β-ODAP content (mg g^{-1})	α-ODAP content (mg g^{-1})	% β-ODAP
LS 001	3.93 ± 0.14	1.14 ± 0.04	78 ± 0.1
LS 002	3.40 ± 0.25	1.15 ± 0.09	75 ± 0.1
LS 003	3.02 ± 0.25	1.12 ± 0.09	73 ± 0.2
LS 004	2.89 ± 0.09	1.05 ± 0.05	73 ± 0.3
LS 005	3.36 ± 0.13	1.19 ± 0.04	74 ± 0.3
LS 006	4.12 ± 0.19	1.22 ± 0.05	77 ± 0.2
LS 007	3.80 ± 0.11	1.24 ± 0.09	75 ± 0.8
LS 008	4.45 ± 0.37	1.50 ± 0.10	75 ± 0.3
LS 009	3.02 ± 0.26	1.06 ± 0.09	74 ± 0.1
LS 010	3.29 ± 0.07	1.02 ± 0.02	76 ± 0.1
LS 011	2.67 ± 0.03	1.00 ± 0.02	73 ± 0.1
LS 012	3.88 ± 0.05	1.29 ± 0.02	75 ± 0.1
LS 013	4.95 ± 0.08	1.41 ± 0.03	78 ± 0.2
LS 014	4.64 ± 0.26	1.43 ± 0.07	76 ± 0.2
LS 015	4.21 ± 0.07	1.27 ± 0.04	77 ± 0.3
LS 016	6.04 ± 0.45	1.84 ± 0.14	77 ± 0.2
LS 017	4.02 ± 0.20	1.06 ± 0.03	79 ± 0.4
LS 018	4.20 ± 0.23	1.37 ± 0.05	75 ± 0.3
LS 019	3.91 ± 0.23	1.18 ± 0.09	77 ± 0.4
LS 020	3.80 ± 0.24	1.32 ± 0.09	74 ± 0.2
LS 021	4.48 ± 0.24	1.79 ± 0.11	71 ± 0.1
LS 022	3.57 ± 0.09	1.07 ± 0.03	77 ± 0.1
LS 023	4.86 ± 0.13	1.81 ± 0.05	73 ± 0.2
LS 024	3.87 ± 0.19	1.41 ± 0.07	73 ± 0.1
LS 025	4.75 ± 0.34	1.31 ± 0.07	78 ± 0.3
LS 026	2.64 ± 0.11	0.92 ± 0.02	74 ± 0.3
LS 027	3.37 ± 0.28	1.24 ± 0.12	73 ± 0.3
LS 028	3.80 ± 0.21	1.10 ± 0.05	78 ± 0.2
LS 029	4.05 ± 0.14	1.36 ± 0.05	75 ± 0.05
LS 030	3.18 ± 0.07	1.00 ± 0.01	76 ± 0.2
LS 031	2.95 ± 0.21	1.10 ± 0.09	73 ± 0.2
LS 032	2.18 ± 0.07	0.84 ± 0.02	72 ± 0.4
LS 033	3.09 ± 0.22	1.13 ± 0.08	73 ± 0.5
LS 034	2.63 ± 0.06	1.02 ± 0.01	72 ± 0.2
LS 035	3.37 ± 0.17	1.19 ± 0.04	74 ± 0.5
LS 036	2.79 ± 0.08	0.99 ± 0.02	74 ± 0.2
LS 037	2.66 ± 0.18	1.06 ± 0.07	71 ± 0.1
LS 038	2.65 ± 0.04	0.89 ± 0.01	75 ± 0.01
LS 039	3.79 ± 0.05	1.30 ± 0.04	74 ± 0.4
LS 040	2.83 ± 0.11	0.98 ± 0.06	74 ± 0.5
LS 041	4.81 ± 0.02	1.43 ± 0.01	77 ± 0.04

LS 042	2.81 ± 0.11	1.05 ± 0.07	73 ± 0.6
LS 043	2.57 ± 0.08	0.94 ± 0.03	73 ± 0.1
LS 044	2.25 ± 0.08	0.91 ± 0.03	71 ± 0.2
LS 045	2.60 ± 0.09	0.87 ± 0.03	75 ± 0.1
LS 046	2.92 ± 0.07	1.09 ± 0.02	73 ± 0.2
LS 047	3.47 ± 0.10	1.20 ± 0.03	74 ± 0.2
LS 048	2.91 ± 0.16	1.13 ± 0.06	72 ± 0.4
LS 049	3.30 ± 0.08	1.18 ± 0.01	74 ± 0.3
LS 050	2.60 ± 0.07	1.06 ± 0.04	71 ± 0.2
LS 051	4.65 ± 0.31	1.37 ± 0.10	77 ± 0.2
LS 052	3.37 ± 0.12	1.22 ± 0.05	73 ± 0.1
LS 053	3.61 ± 0.05	1.09 ± 0.02	77 ± 0.3
LS 054	4.35 ± 0.23	1.58 ± 0.08	73 ± 0.2
LS 055	3.54 ± 0.20	1.43 ± 0.07	71 ± 0.2
LS 056	3.37 ± 0.19	1.30 ± 0.07	72 ± 0.1
LS 057	3.46 ± 0.13	1.07 ± 0.06	76 ± 0.7
LS 058	3.21 ± 0.11	1.46 ± 0.06	69 ± 0.2
LS 059	1.94 ± 0.11	0.77 ± 0.04	72 ± 0.3
LS 060	3.10 ± 0.15	1.03 ± 0.04	75 ± 0.1
LS 061	3.02 ± 0.02	1.05 ± 0.002	74 ± 0.1
LS 062	2.57 ± 0.02	0.97 ± 0.01	73 ± 0.3
LS 063	2.98 ± 0.16	1.05 ± 0.06	74 ± 0.1
LS 064	3.19 ± 0.18	0.97 ± 0.04	77 ± 0.3
LS 065	3.68 ± 0.15	1.43 ± 0.02	72 ± 0.7
LS 066	3.07 ± 0.09	0.88 ± 0.02	78 ± 0.1
LS 067	3.28 ± 0.07	1.31 ± 0.04	71 ± 0.2
LS 068	3.06 ± 0.01	1.11 ± 0.02	73 ± 0.3
LS 069	3.72 ± 0.06	1.02 ± 0.01	79 ± 0.2
LS 070	3.53 ± 0.11	0.90 ± 0.02	80 ± 0.2
LS 071	3.92 ± 0.29	1.29 ± 0.10	75 ± 0.03
LS 072	2.53 ± 0.10	0.74 ± 0.02	77 ± 0.2
LS 073	2.93 ± 0.11	0.97 ± 0.04	75 ± 0.4
LS 074	3.65 ± 0.05	0.80 ± 0.00	82 ± 0.1
LS 075	2.11 ± 0.05	0.67 ± 0.01	76 ± 0.2
LS 076	4.72 ± 0.11	1.22 ± 0.03	79 ± 0.3
LS 078	4.00 ± 0.05	0.73 ± 0.02	85 ± 0.1
LS 079	3.97 ± 0.15	0.75 ± 0.01	84 ± 0.3
LS 080	3.89 ± 0.13	0.81 ± 0.04	83 ± 0.1
LS 081	4.99 ± 0.06	0.80 ± 0.02	86 ± 0.2
LS 082	4.35 ± 0.10	0.78 ± 0.07	85 ± 0.9
LS 083	2.82 ± 0.08	0.79 ± 0.01	78 ± 0.3
LS 084	4.03 ± 0.29	1.21 ± 0.08	77 ± 0.3
LS 085	2.08 ± 0.07	0.71 ± 0.02	75 ± 0.2
LS 086	3.27 ± 0.14	1.02 ± 0.06	76 ± 0.4
LS 087	3.35 ± 0.18	0.85 ± 0.05	80 ± 0.3
LS 088	4.56 ± 0.29	1.17 ± 0.08	80 ± 0.5

LS 091	4.43 ± 0.18	1.38 ± 0.04	76 ± 0.2
LS 093	4.29 ± 0.15	1.28 ± 0.03	77 ± 0.2
LS 094	3.34 ± 0.19	0.75 ± 0.04	82 ± 0.1
LS 095	3.62 ± 0.09	0.91 ± 0.03	80 ± 0.1
LS 096	4.21 ± 0.06	1.50 ± 0.02	74 ± 0.1
LS 097	5.42 ± 0.17	1.53 ± 0.05	78 ± 0.01
LS 098	3.02 ± 0.30	0.98 ± 0.08	75 ± 0.4
LS 099	3.86 ± 0.17	0.69 ± 0.04	85 ± 0.3
LS 101	3.40 ± 0.05	0.74 ± 0.0 ±1	82 ± 0.3
LS 103	2.79 ± 0.18	0.79 ± 0.04	78 ± 0.7
LS 104	0.45 ± 0.02	0.16 ± 0.005	74 ± 0.4
LS 105	2.49 ± 0.03	0.89 ± 0.01	74 ± 0.2
LS 106	2.54 ± 0.07	0.92 ± 0.03	73 ± 0.2
LS 107	4.32 ± 0.05	1.38 ± 0.02	76 ± 0.1
LS 108	4.57 ± 0.04	1.03 ± 0.01	82 ± 0.1
LS 109	3.95 ± 0.20	1.33 ± 0.05	75 ± 0.4
LS 112	3.26 ± 0.06	0.56 ± 0.02	85 ± 0.1
LS 113	4.69 ± 0.27	0.84 ± 0.04	85 ± 0.2
LS 114	2.25 ± 0.11	0.89 ± 0.06	72 ± 0.5
LS 115	2.95 ± 0.03	1.01 ± 0.01	74 ± 0.03
LS 110*	1.32 ± 0.06	0.21 ± 0.02	86 ± 0.5
LS 118*	0.94 ± 0.03	0.37 ± 0.01	72 ± 0.4

Table S2. Germplasm bank, geographical origin and seed size, colour and varietal group characterization. n/a: not applicable. (*) *Lathyrus cicera* samples.

Sample	Donor	Germplasm bank	Geographical origin	Seed size	Seed Colour	Varietal group
LS 001	USDA-ARS	PI163293	India	Small	Dark	Indian
LS 002	USDA-ARS	PI165528	India	Small	Dark	Indian
LS 003	USDA-ARS	PI170469	Turkey	Small	Dark	Indian
LS 004	USDA-ARS	PI170470	Turkey	Small	Dark	Indian
LS 005	USDA-ARS	PI172930	Turkey	Small	Dark	Indian
LS 006	INIA-CRF	BGE1490	Spain	Large	Light	Mediterranean
LS 007	USDA-ARS	PI179939	India	Small	Dark	Indian
LS 008	USDA-ARS	PI180848	Turkey	Small	Dark	Indian
LS 009	USDA-ARS	PI193544	Ethiopia	Small	Dark	Indian
LS 010	USDA-ARS	PI195603	Ethiopia	Small	Dark	Indian
LS 011	USDA-ARS	PI195993	Ethiopia	Small	Dark	Indian
LS 012	USDA-ARS	PI195998	Ethiopia	Small	Dark	Indian
LS 013	USDA-ARS	PI196001	Ethiopia	Small	Dark	Indian
LS 014	USDA-ARS	PI220176	Afghanistan	Small	Dark	Indian
LS 015	USDA-ARS	PI221467	Afghanistan	Small	Dark	Indian
LS 016	USDA-ARS	PI223269	Afghanistan	Small	Dark	Indian
LS 017	USDA-ARS	PI226948	Ethiopia	Small	Dark	Indian
LS 018	USDA-ARS	PI230345	Iran	Small	Dark	Indian
LS 019	USDA-ARS	PI232923	Hungary	Large	Light	Mediterranean
LS 020	USDA-ARS	PI244756	Ethiopia	Small	Dark	Indian
LS 021	USDA-ARS	PI251413	Iran	Small	Dark	Indian
LS 022	INIA-CRF	BGE15746	Spain	Large	Light	Mediterranean
LS 023	USDA-ARS	PI257589	Ethiopia	Small	Dark	Indian
LS 024	USDA-ARS	PI268478	Afghanistan	Small	Dark	Indian
LS 025	USDA-ARS	PI269921	Pakistan	Small	Dark	Indian
LS 026	USDA-ARS	PI283547	France	Small	Dark	Indian
LS 027	USDA-ARS	PI283550	Former Soviet Union	Small	Light	Intermediate

LS 028	USDA-ARS	PI283553	Italy	Small	Light	Intermediate
LS 029	USDA-ARS	PI283554	Former Soviet Union	Small	Light	Intermediate
LS 030	IAS-CSIC	LISA	Spain	Large	Light	Mediterranean
LS 031	USDA-ARS	PI283560	Morocco	Small	Light	Intermediate
LS 032	USDA-ARS	PI283561	Greece	Small	Light	Intermediate
LS 033	USDA-ARS	PI227847	Iran	Small	Dark	Indian
LS 034	USDA-ARS	PI283564	Sudan	Small	Dark	Indian
LS 035	USDA-ARS	PI283565	Morocco	Small	Dark	Indian
LS 036	USDA-ARS	PI283566	Morocco	Small	Dark	Indian
LS 037	USDA-ARS	PI283568	Hungary	Small	Light	Intermediate
LS 038	USDA-ARS	PI283569	Libya	Large	Light	Mediterranean
LS 039	USDA-ARS	PI283570	Algeria	Small	Light	Intermediate
LS 040	USDA-ARS	PI283572	Cyprus	Small	Light	Intermediate
LS 041	INIA-CRF	BGE1490	Spain	Large	Light	Mediterranean
LS 042	USDA-ARS	PI283580	Cyprus	Large	Light	Mediterranean
LS 043	USDA-ARS	PI283582	Italy	Small	Dark	Indian
LS 044	USDA-ARS	PI283583	Italy	Small	Dark	Indian
LS 045	USDA-ARS	PI283586	Cyprus	Small	Light	Intermediate
LS 046	USDA-ARS	PI283592	Cyprus	Small	Light	Intermediate
LS 047	USDA-ARS	PI283593	Czechoslovakia	Small	Light	Intermediate
LS 048	USDA-ARS	PI283595	Poland	Small	Light	Intermediate
LS 049	USDA-ARS	PI283596	Afghanistan	Small	Dark	Indian
LS 050	USDA-ARS	PI283597	Tunisia	Large	Light	Mediterranean
LS 051	USDA-ARS	PI286531	India	Small	Dark	Indian
LS 052	USDA-ARS	PI317440	Afghanistan	Small	Dark	Indian
LS 053	INIA-CRF	BGE15746	Spain	Large	Light	Mediterranean
LS 054	USDA-ARS	PI317443	Afghanistan	Small	Dark	Indian
LS 055	USDA-ARS	PI358600	Ethiopia	Small	Dark	Indian
LS 056	USDA-ARS	PI358601	Ethiopia	Small	Dark	Indian
LS 057	USDA-ARS	PI370600	Former Serbia and Montenegro	Small	Dark	Indian
LS 058	USDA-ARS	PI380888	Iran	Small	Dark	Indian

LS 059	USDA-ARS	PI391430	India	Small	Dark	Indian
LS 060	USDA-ARS	PI391431	India	Small	Dark	Indian
LS 061	USDA-ARS	PI391432	India	Small	Dark	Indian
LS 062	ICARDA	ACC170	n/a	Small	Dark	Indian
LS 063	USDA-ARS	PI422521	Hungary	Small	Dark	Indian
LS 064	USDA-ARS	PI422526	Hungary	Large	Light	Mediterranean
LS 065	INIA-CRF	BGE17184	Spain	Large	Light	Mediterranean
LS 066	USDA-ARS	PI422532	Former Soviet Union	Small	Light	Intermediate
LS 067	USDA-ARS	PI422533	Former Soviet Union	Small	Dark	Indian
LS 068	USDA-ARS	PI422535	Turkey	Large	Dark	Intermediate
LS 069	USDA-ARS	PI422536	Italy	Small	Light	Intermediate
LS 070	USDA-ARS	PI422537	Hungary	Large	Light	Mediterranean
LS 071	USDA-ARS	PI422538	Former Soviet Union	Small	Light	Intermediate
LS 072	USDA-ARS	PI422540	Italy	Small	Light	Intermediate
LS 073	USDA-ARS	PI422541	Former Soviet Union	Small	Light	Intermediate
LS 074	USDA-ARS	PI426880	Pakistan	Small	Dark	Indian
LS 075	ICARDA	ACC190	n/a	Small	Dark	Indian
LS 076	INIA-CRF	BGE1490	Spain	Large	Light	Mediterranean
LS 078	USDA-ARS	PI426884	Pakistan	Small	Dark	Indian
LS 079	USDA-ARS	PI426886	Pakistan	Small	Dark	Indian
LS 080	USDA-ARS	PI426890	Pakistan	Small	Dark	Indian
LS 081	USDA-ARS	PI426894	Pakistan	Small	Dark	Indian
LS 082	USDA-ARS	PI426897	Pakistan	Small	Dark	Indian
LS 083	USDA-ARS	PI442793	India	Small	Dark	Indian
LS 084	USDA-ARS	PI513244	Pakistan	Small	Dark	Indian
LS 085	ICARDA	ACC192	n/a	Small	Dark	Indian
LS 086	INIA-CRF	BGE15746	Spain	Large	Light	Mediterranean
LS 087	USDA-ARS	PI543071	Pakistan	Small	Dark	Indian
LS 088	USDA-ARS	PI568190	Turkey	Small	Dark	Indian
LS 091	USDA-ARS	PI577139	Bulgaria	Small	Light	Intermediate
LS 093	USDA-ARS	PI667238	Greece	Small	Light	Intermediate

LS 094	USDA-ARS	PI667247	Pakistan	Small	Dark	Indian
LS 095	USDA-ARS	PI667250	Albania	Large	Light	Mediterranean
LS 096	USDA-ARS	PI667251	Poland	Large	Light	Mediterranean
LS 097	USDA-ARS	PI667252	Tajikistan	Small	Dark	Indian
LS 098	USDA-ARS	PI667263	Georgia	Small	Light	Intermediate
LS 099	INIA-CRF	BGE17184	Spain	Large	Light	Mediterranean
LS 101	INIA-CRF	BGE29748	Spain	Large	Light	Mediterranean
LS 103	Fernand Lambein	RAIPUR	from India	Small	Dark	Indian
LS 104	Fernand Lambein (Breeding line Clayton Campbell, AgriFoodCanada)	LS87124	Canada	Small	Light	Intermediate
LS 105	INIA-CRF	BGE15746	Spain	Large	Light	Mediterranean
LS 106	INIA-CRF	BGE15746	Spain	Large	Light	Mediterranean
LS 107	INIA-CRF	BGE1490	Spain	Large	Light	Mediterranean
LS 108	INIA-CRF	BGE1490	Spain	Large	Light	Mediterranean
LS 109	INIA-CRF	BGE1490	Spain	Large	Light	Mediterranean
LS 112	IFVC	SITNICA	Serbia	Small	Dark	Indian
LS 113	IFVC	STUDENICA	Serbia	Large	Light	Mediterranean
LS 114	INIA-CRF	BGE15746	Spain	Large	Light	Mediterranean
LS 115	INIA-CRF	BGE29748	Spain	Large	Light	Mediterranean
LS 110*	HAO-DEMETER	RHODOS	Greece	n/a	n/a	n/a
LS 118*	INIA-CRF	BGE8277	Spain	n/a	n/a	n/a
